# The effects of repeated inhaler device handling education in COPD patients: a prospective cohort study

**DOI:** 10.1038/s41598-020-76961-y

**Published:** 2020-11-12

**Authors:** June Hong Ahn, Jin Hong Chung, Kyeong-Cheol Shin, Hyun Jung Jin, Jong Geol Jang, Mi Suk Lee, Kwan Ho Lee

**Affiliations:** Division of Pulmonology and Allergy, Department of Internal Medicine, Yeungnam University Medical Center, College of Medicine, Yeungnam University, 170 Hyeonchung-ro, Namgu, 42415 Daegu Republic of Korea

**Keywords:** Diseases, Health care, Medical research

## Abstract

Inhaler education for chronic obstructive pulmonary disease (COPD) patients improves inhaler technique and adherence. However, the effects of such education on the quality of life and inhaler satisfaction remain unclear. Here, we evaluated inhaler handling and adherence, and changes in quality of life and inhaler satisfaction, after repeated education for COPD patients. We prospectively enrolled COPD patients who had used inhalers for over 1 month and evaluated the effects of repeated education. Three visits were made over 6 months; an advanced practice nurse evaluated inhaler technique and adherence, and instructed the patients in inhaler technique during face-to-face sessions. Inhaler technique and adherence were assessed at every visits, and the modified Medical Research Council (mMRC) test, COPD Assessment Test (CAT), EuroQol-5D (EQ-5D), Patient Health Questionnaire (PHQ-9), and Feeling of Satisfaction with Inhaler questionnaire (FSI-10) were administered before (visit 1) and after two educational sessions (visit 3). A total of 261 COPD patients (308 inhalers) were included. Education significantly reduced the proportion of critical errors after two educational sessions (visit 3), from 43.2 to 8.8% (p < 0.001). The proportion of highly compliant patients increased after two visits, from 81.6% to 87.7% (p = 0.005). The FSI-10 score improved significantly after education, from 44.36 ± 4.69 to 47.64 ± 4.08 (p < 0.001); the scores on the other instruments (mMRC, CAT, EQ-5D, and PHQ-9) did not improve. Repeated face-to-face inhaler education by an advanced practice nurse significantly improved inhaler satisfaction, technique, and adherence. However, inhaler education did not significantly improve quality of life.

## Introduction

Chronic obstructive pulmonary disease (COPD) exhibits many different phenotypes, and the prevalence ranged from 12.9 to 17.2% in the Korean National Health and Nutritional Examination Survey II (KNHANES II)^1,2^. Correct inhaler use is important: incorrect use is associated with an increased risk of acute exacerbation, hospital admission, emergency room visits, and a need for antimicrobials and oral steroids^3–5^. However, in the real world, inhaler mishandling and poor adherence are very common, despite the fact that most COPD patients receive education on inhaler use^3,5,6^. Many studies have shown that education reduces inhaler mishandling, significantly improving inhaler technique^6–9^.

Quality of life refers to satisfaction or happiness in aspects of life when an individual is affected by their health^10,11^. Quality of life of COPD patients was lower than that of the general population. High severity of COPD, depression, and osteoporosis were associated with lower quality of life in Korean COPD patients^11^. Patient satisfaction with inhaler device is associated with patient adherence and clinical outcomes. In a large, multinational, cross-sectional, real-world survey with COPD patients, significant association was reported between inhaler satisfaction and treatment adherence. Furthermore, there was a direct association between inhaler satisfaction and fewer COPD exacerbations^12^.

Few studies have examined the association between inhaler education and quality of life^6,13–15^; no study has explored the relationship between inhaler education and inhaler satisfaction. Thus, we evaluated inhaler handling and adherence, and changes in quality of life and inhaler satisfaction, after repeated education for COPD patients.

## Materials and methods

### Study design and subjects

**Figure 1:**
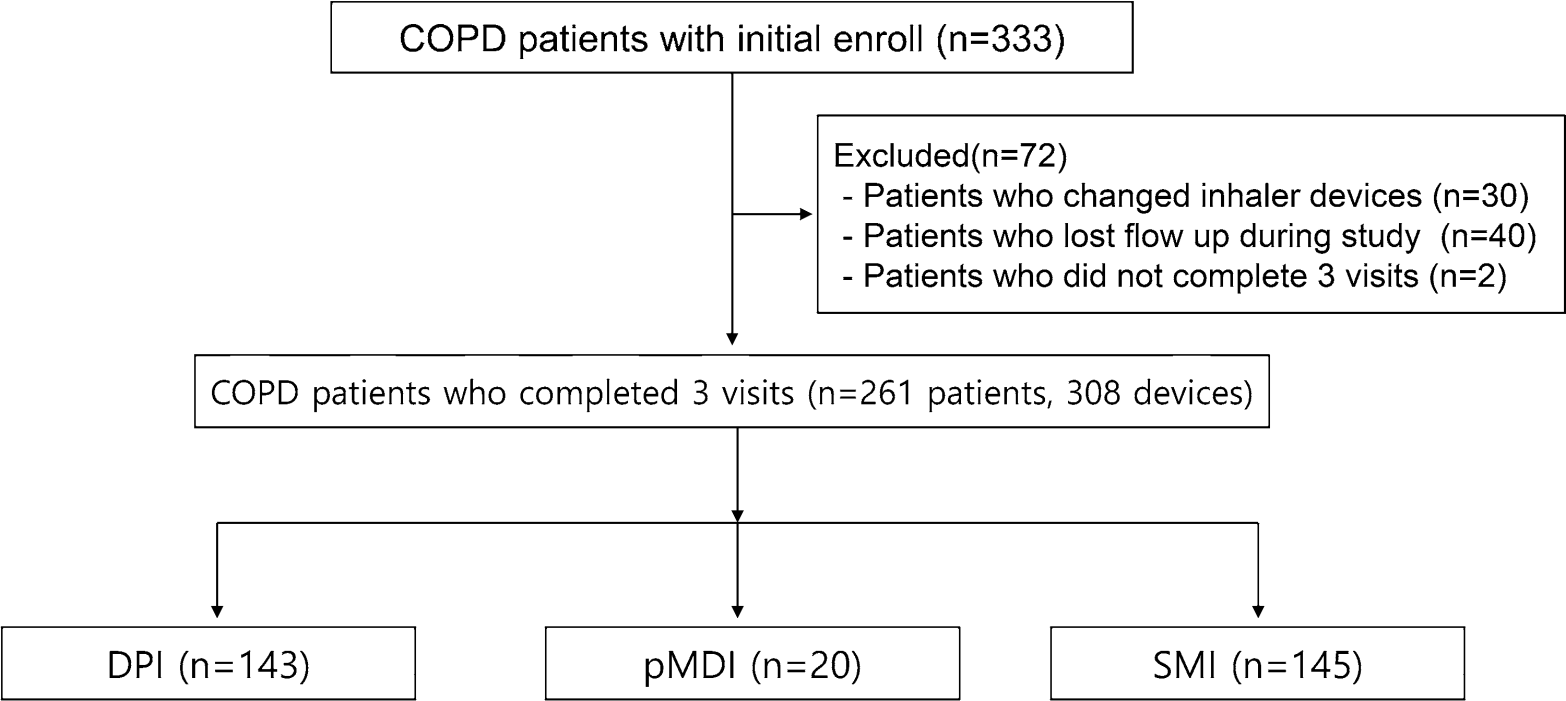
Flow diagram of the study subjects. *COPD* chronic obstructive pulmonary disease, *DPI* dry powder inhaler, *pMDI* pressurized metered-dose inhaler, *SMI* soft mist inhaler.

This prospective study was conducted in the pulmonology outpatient department of the Regional Center for Respiratory Diseases, Yeungnam University Hospital (a tertiary university hospital in Daegu, South Korea) from January 2018 to May 2019. Patients aged over 40 years and diagnosed with COPD were initially enrolled, and all those who had used inhalers of any kind for more than 1 month were recruited to the study. The intervention included three visits over 6 months; follow-up visits were performed every 3 months. In total, 72 patients were excluded for the following reasons: inhaler device changed during the study period (n = 30); lost to follow-up (n = 40); and did not complete the three visits (n = 2). COPD patients who completed 3 visits and maintained the same inhaler device during study period were finally analyzed. Finally, 261 patients using 308 inhalers were included (Fig. 1). The inhalers included the Turbuhaler, Breezhaler, Ellipta, Diskus, Genuair, Respimat, and pressurized metered dose inhalers (pMDI) models. We excluded patients using a pMDI with a spacer, using other inhalers, those with advanced cancer, and pregnant females.

### Patient visits

During the study, patients who agreed to the study were enrolled among all COPD patients who visited our respiratory outpatient clinic. The intervention included three visits over 6 months; follow-up visits were performed every 3 months. All patients had undergone pulmonary function tests within the 3 months prior to enrolment. At visit 1 (baseline), written informed consent was obtained from all patients. A general questionnaire exploring age, sex, body mass index, smoking status, COPD duration, previous inhaler education, previous COPD education, and educational level was administered. The modified Medical Research Council test (mMRC)^16^, the COPD Assessment Test (CAT)^17^, the Mini-Mental State Examination (MMSE)^18^, the EuroQol-5D (EQ-5D) instrument^19^, the Patient Health Questionnaire (PHQ-9)^20^, to assess the quality of life and the Feeling of Satisfaction with Inhaler questionnaire (FSI-10)^21,22^ were administered at the first visit. All questionnaires were available free online. An advanced practice nurse assessed inhaler technique and adherence, and delivered face-to-face training using the “teach-back” technique, in which the nurse says: “Can you show me what I showed you and explain it to me?” “Teach-back” is a technique that requires patients to explain or demonstrate their skills back after training^23^. Repetitive training using the “teach-back” technique was conducted in visit 1 until the patient fully understands the inhaler device and fully explain the operation of the inhaler. At visits 2 and 3, the nurse re-assessed inhaler technique and adherence and delivered face-to-face training using “teach-back” technique if any error was apparent. At visit 3, we re-administered the mMRC, CAT, EQ-5D, and PHQ-9, to assess changes in quality of life, and the FSI-10.

### Data collection and definitions

An advanced practice nurse specializing in inhaler education performed all of the interviews and training sessions^5^. The nurse was educated by our COPD specialists and had trained COPD patients in inhaler techniques for 3 years. Critical errors were defined as errors seriously compromising drug delivery to the lung. We created a standardized checklist of inhaler use critical steps by reference to the review literature^24^. The critical errors are listed in Table 2. Adherence was self-reported and graded as good, partial, or poor, according to whether the entire daily dose was taken, the daily dose (frequency or amount) taken was more or less than required, and the medication was taken only as needed or not at all, respectively^25^. The FSI-10 (10 questions) is a validated self-administered questionnaire evaluating patient satisfaction with their inhaler^21,22^. The answer options range from “hardly at all” (score of 1 on a 5-point Likert scale) to 5 “very” (score of 5); the total score thus ranges from 10 to 50; higher scores indicate better satisfaction. Inhaler convenience, maintenance, portability, and “feel” are all assessed by the FSI-10.

### Statistical analysis

Continuous variables are expressed as means ± standard deviations (SDs) and were compared using Student’s *t*-test or the Mann–Whitney U test. Categorical variables were compared using the chi-squared test or Fisher’s exact test. In all analyses, a two-tailed p-value < 0.05 was considered to indicate statistical significance. All statistical analyses were performed using SPSS software (ver. 24.0; SPSS Inc., Chicago, IL, USA). A prospective power calculation indicated that an overall sample size of 220 was required to evaluate the efficacy of education (95% power, α = 0.05, effect size = 0.3). To allow for dropout, we sought to enroll 260 patients^26^.

### Ethics approval and consent to participate

This study was conducted in accordance with all relevant tenets of the Declaration of Helsinki. The protocol was reviewed and approved by the institutional review board of our hospital (Yeungnam University Hospital Institutional Review Board 2017-09-012-001). Written informed consent was obtained from all patients.

## Results

### Baseline characteristics

Patient baseline characteristics are listed in Table 1. The mean age was 69.8 years and males predominated (93.5%). The mean body mass index was 23.5 kg/m^2^ and the mean COPD duration was 3.6 years. In total, 47 (18.0%) patients were current smokers and 179 (68.6%) were ex-smokers; 95.4% had received previous education on COPD and inhaler handling. One-third of the patients were poorly educated. Most exhibited mild-to-moderate airflow limitation (63.5 ± 17.5% of the predicted forced expiratory volume in 1 s [FEV_1_]). The mean mMRC and CAT scores were 1.3 ± 0.9 and 9.9 ± 5.6 respectively. The mean MMSE score was 29.3 ± 1.6.

**Table 1:** Baseline characteristics of the COPD patients.

Variable	N = 261
Age (years)	69.8 ± 7.7
Male, n (%)	244 (93.5)
Body mass index (kg/m^2^)	23.5 ± 3.5
Smoking status	
Never-smoker	35 (13.4)
Ex-smoker	179 (68.6)
Current smoker	47 (18.0)
COPD duration (years)	3.6 ± 4.3
Multiple inhaler devices (≥ 2 devices)	47 (18.0)
Previous education on COPD	249 (95.4)
Previous education on how to handle an inhaler	249 (95.4)
Educational level	
Low (≤ 6 years) Higher (> 6 years)	99 (37.9) 162 (62.1)
FEV_1_/FVC (%)	58.6 ± 13.7
Percentage predicted FEV_1_	63.5 ± 17.5
Percentage predicted DLCO (n = 258)	68.4 ± 19.5
GOLD stage	
I, II	204 (78.1)
III, IV	57 (21.9)
mMRC score	1.3 ± 0.9
CAT score	9.9 ± 5.6
MMSE score (n = 258)	29.3 ± 1.6
Frequent exacerbations in the prior year	65 (24.9)

Data are presented as means ± standard deviations (ranges) or numbers (percentages).

*CAT* COPD assessment test, *COPD* chronic obstructive pulmonary disease, *DLCO* diffusion capacity for carbon monoxide, *FEV*_*1*_ forced expiratory volume in 1 s, *FVC* forced vital capacity, *GOLD* Global Initiative for Chronic Obstructive Lung Disease, *mMRC* modified Medical Research Council, *MMSE* Mini-Mental State Examination.

### Inhaler use/adherence before and after education

**Figure 2:**
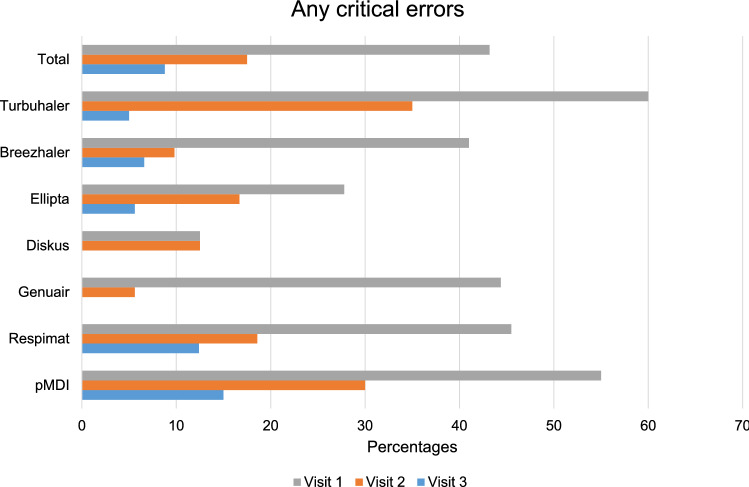
Inhaler use critical errors before and after education.

**Figure 3:**
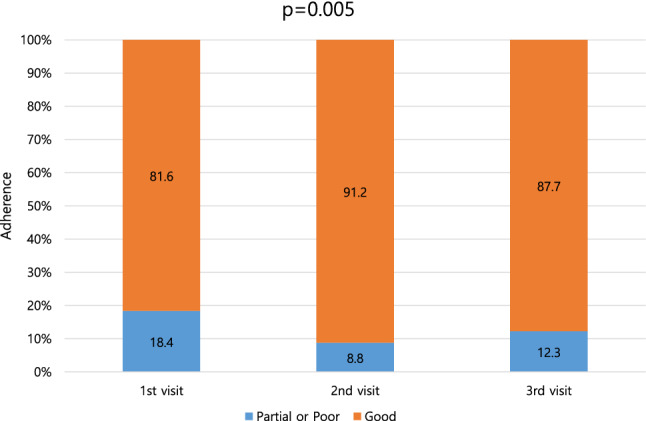
Adherence before and after education.

A total of 261 COPD patients using 308 inhaler devices were enrolled. The percentages of patients exhibiting at least one critical error during inhaler use, before and after education, are listed in Fig. 2. At visit 1, 43.2% (133/308) showed at least one critical error. After two educational visits, these values fell to 8.8% (27/308); education improved the use of all included inhalers (Table 2). All critical errors were reduced after repeated education. In terms of adherence, the proportion of good compliers increased after two educational sessions, from 81.6 to 87.7% (p = 0.005; Fig. 3).

**Table 2:** Critical errors for each inhaler device before and after education.

Critical steps of inhalation technique	Any critical errors		P-value
	Before education (Visit 1)	After education (Visit 3)	
**Turbuhaler, n = 20**			
Open the device correctly	0 (0)	0 (0)	
Prime with device upright	9 (45.0)	1 (5.0)	0.003
Seal lips around mouthpiece during inhalation	0 (0)	0 (0)	
Inhale forcefully or deeply	4 (20.0)	0 (0)	0.106
**Breezhaler, n = 61**			
Open the device correctly	0 (0)	0 (0)	
Place capsule in the chamber	1 (1.6)	0 (0)	1.000
Close the mouthpiece	5 (2.5)	0 (0)	0.057
Press button to pierce the capsule	13 (21.3)	0 (0)	< 0.001
Seal lips around mouthpiece during inhalation	0 (0)	0 (0)	
Inhale forcefully or deeply	16 (26.2)	4 (6.6)	0.003
Remove capsule and check for powder residue	6 (9.8)	0 (0)	0.027
**Ellipta, n = 36**			
Open the device correctly	2 (5.6)	1 (2.8)	1.000
Seal lips around mouthpiece during inhalation	0 (0)	0 (0)	
Inhale forcefully or deeply	8 (22.2)	1 (2.8)	0.028
**Diskus, n = 8**			
Open the device correctly	0 (0)	0 (0)	
Pull the lever fully back	1 (12.5)	0 (0)	1.000
Seal lips around mouthpiece during inhalation	0 (0)	0 (0)	
Inhale forcefully or deeply	0 (0)	0 (0)	
**Genuair, n = 18**			
Open the device correctly	0 (0)	0 (0)	
Hold the inhaler horizontally (green button facing upwards) for priming	3 (16.7)	0 (0)	0.229
Seal lips around mouthpiece during inhalation	0 (0)	0 (0)	
Inhale forcefully or deeply	6 (33.3)	0 (0)	0.019
**Respimat, n = 145**			
Twist the base one half-turn	28 (19.3)	4 (2.8)	< 0.001
Open the device correctly	27 (18.6)	4 (2.8)	< 0.001
Seal lips around mouthpiece during inhalation	10 (6.9)	2 (1.4)	0.035
Synchronize actuation and inhalation	36 (24.8)	12 (8.3)	< 0.001
Inhale slowly and deeply	39 (26.9)	13 (9.0)	< 0.001
**pMDI, n = 20**			
Open the device correctly	0 (0)	0 (0)	
Shake well (suspension formulations only)	3 (15.0)	0 (0)	0.231
Keep inhaler upright	3 (15.0)	1 (5.0)	0.605
Seal lips around mouthpiece during inhalation	0 (0)	0 (0)	
Synchronize actuation and inhalation	5 (25.0)	1 (5.0)	0.182
Inhale slowly and deeply	6 (30.0)	2 (10.0)	0.235

Data are presented as numbers (percentages).

### Quality of life before and after education

We compared the quality of life before (visit 1) and after (visit 3) education. The scores on the mMRC, CAT, EQ-5D, or PHQ-9 did not improve significantly (Table 3). Each parameters in the EQ-5D domain did not show any significant improvement after education (Table 4).

**Table 3:** Quality of life before and after education.

	Before education (n = 261)	After education (n = 261)	P-value
mMRC	1.31 ± 0.87	1.27 ± 0.96	0.617
CAT	9.92 ± 5.56	10.78 ± 6.44	0.102
EQ-5D	0.86 ± 0.11	0.85 ± 0.14	0.292
PHQ-9	1.14 ± 2.30	1.56 ± 3.16	0.101

Data are presented as means ± standard deviations (ranges).

*CAT* COPD assessment test, *EQ-5D* EuroQol-5D, *FSI-10* Feeling of Satisfaction with Inhaler questionnaire, *mMRC* modified Medical Research Council, *PHQ-9* Patient Health Questionnaire.

**Table 4:** EQ-5D domains before and after education.

EQ-5D domains	Education status	EQ-5D level			P value
		No problem	Some problem	Severe problem	
Mobility	Before education	161 (61.7)	98 (37.5)	2 (0.8)	0.791
	After education	165 (63.2)	92 (35.2)	4 (1.5)	
Self-care	Before education	216 (82.8)	44 (16.9)	1 (0.4)	0.606
	After education	222 (85.1)	35 (13.4)	4 (1.5)	
Usual activities	Before education	193 (73.9)	67 (25.7)	1 (0.4)	0.002
	After education	160 (61.3)	98 (37.5)	3 (1.1)	
Pain/discomfort	Before education	194 (74.3)	65 (24.9)	2 (0.8)	0.272
	After education	183 (70.1)	75 (28.7)	3 (1.1)	
Anxiety/depression	Before education	195 (74.7)	63 (24.1)	3 (1.1)	0.846
	After education	193 (73.9)	65 (24.9)	3 (1.1)	

Data are presented as numbers (percentages).

*EQ-5D* EuroQol-5D.

### Inhaler satisfaction

Table 5 shows the inhaler satisfaction scores before and after education. Scores on all 10 items of the FSI-10 (all 10 items p < 0.001 respectively), and the overall score (44.36 ± 4.69 to 47.64 ± 4.08, p < 0.001), improved significantly after two educational sessions.

**Table 5:** Inhaler satisfaction before and after education.

	Before education (n = 261)	After education (n = 261)	P-value
FSI-10	44.36 ± 4.69	47.64 ± 4.08	< 0.001
Easy to learn how to use the inhaler	4.36 ± 0.70	4.80 ± 0.55	< 0.001
Easy to prepare the inhaler for use	4.48 ± 0.58	4.80 ± 0.52	< 0.001
Easy to use the inhaler	4.51 ± 0.67	4.77 ± 0.55	< 0.001
Easy to keep the inhaler clean and in good working condition	4.50 ± 0.60	4.85 ± 0.39	< 0.001
Easy to continue normal activities with the use of the inhaler	4.38 ± 0.68	4.69 ± 0.65	< 0.001
Inhaler fits my lips comfortably	4.51 ± 0.66	4.84 ± 0.46	< 0.001
Easy to use in terms of size and weight	4.65 ± 0.49	4.87 ± 0.40	< 0.001
Easy to carry the inhaler	4.20 ± 0.74	4.49 ± 0.67	< 0.001
I feel that I am using the inhaler correctly	4.45 ± 0.77	4.78 ± 0.57	< 0.001
Overall, I am satisfied with the inhaler	4.33 ± 0.73	4.75 ± 0.60	< 0.001

Data are presented as means ± standard deviations (ranges).

*FSI-10* Feeling of Satisfaction with Inhaler questionnaire.

## Discussion

Of 261 COPD patients using 308 inhalers, at least one critical error during 133 (43.2%) uses at visit 1. After two “teach-back” educational sessions, those values changed 27 (8.8%), irrespective of inhaler type. The proportion of patients exhibiting good adherence also increased, as did inhaler satisfaction, but not the quality of life.

So far, studies on the effects of inhaler education on quality of life is controversial. While some studies reported positive associations between inhaler educational interventions and quality of life^13,15,27–30^, others did not^6,14^. Two studies showing positive associations evaluated the short-term (1–3 months) effects of education^13,27^; the other two studies enrolled only asthma patients^29,30^. In our study, the mMRC, CAT, EQ-5D, or PHQ-9 instruments revealed no relationship between education and improved quality of life. The characteristics of our population (COPD patients only), and the relatively long interval before measurement of outcomes (6 months) may explain the lack of an association between education and improved quality of life. Although certain subgroups of patients may be expected to enjoy a better quality of life after inhaler education, more research is needed to confirm this.

Satisfaction with inhaler is defined as how satisfied patients’ are with their inhaler devices regarding ease and convenient to use. Inhaler satisfaction is very important part of the treatment with chronic airway diseases, enhancing both adherence and disease control^12,31^. Inhaler satisfaction differs between different inhaler devices in asthma and COPD patients^21,22^. Previous study showed that patients with asthma were significantly more satisfied with the inhaler than patients with COPD. Younger age, good disease control, previous inhaler training, and good adherence were associated with high inhaler satisfaction levels^32^. We found that repeated education significantly improved satisfaction (on all 10 FSI-10 items) in COPD patients. Inhaler satisfaction improvement can affect various clinical outcomes in the long run. However, not much is known about the relationship between inhaler satisfaction improvement and clinical outcomes. Our study has proven the relationship between inhaler education and inhaler satisfaction, and future studies whether there is a correlation between an improved FSI-10 score and better disease control are imperative.

The GOLD 2019 guidelines state that, after reviewing the symptoms and determining the dyspnea and exacerbation status, inhaler technique/adherence should be repeatedly assessed; drug potency is irrelevant if the drug is not delivered properly^33^. Many studies found that educational interventions attenuated inhaler errors and improved adherence in patients with airway diseases^6,7,13,24,27,34^. Repeated education was the optimal approach. Most studies were performed in asthma patients^7,35,36^. Some studies enrolled COPD patients^8,37,38^, but most of the educational programs were brief. In three programs, three educational visits were scheduled at 2-week intervals, or according to a 1-month program^27,37,38^. One study assessed changes in inhaler technique at 4–6 weeks after education^8^. We scheduled three educational visits at 3-month intervals and analyzed the outcomes at 6 months. Our study is unique and has strength in that it included relatively long-term evaluations (6 months) after repeated education of COPD patients, and clearly shows the effectiveness of education on inhaler technique and adherence for a relatively long period (3 months) after one session of education.

Critical errors were common (all inhaler types) at visit 1. Among DPI users, Turbuhaler, Breezhaler, and Genuair users made more critical errors than Diskus and Ellipta users. After two educational sessions, the critical error rate was less than 10% among the DPI users. Those using the Respimat and pMDIs made more critical errors than the DPI users at visit 1. Education decreased the initial rate of critical errors of the Respimat and pMDI users to 10%. Although the improvements differed somewhat among the devices, all critical error rates fell.

One large real-world study assessed 2935 COPD patients using 3393 devices; critical errors were divided into dose preparation and delivery errors^3^. Dose preparation errors were common in Respimat and Turbuhaler users, and dose delivery errors in Respimat and pMDI users; our findings were similar. Dose preparation errors were commonly observed in Turbuhaler users (failure to prime with the device upright, 45.0%), Breezhaler users (failure to press the button that pierces the capsule, 21.3%), Genuair users (failure to hold the inhaler horizontally for priming, 16.7%) and Respimat users (failure to twist the base by one half-turn, 19.3%). Dose delivery errors were more common in Respimat and pMDI users, and included failure to synchronize actuation and inhalation (24.8% and 25.0%, respectively) and failure to inhale slowly and deeply (26.9 and 30.0%, respectively). All critical error rates fell after two educational interventions.

Our work had certain limitations. First, this was a single-center study lacking a control group, so selection bias was inevitable. Inhaler use assessment and education are essential components of COPD management. so it would have been unethical to include a control group. Therefore, we compared several parameters before and after the educational intervention. Also, 40 patients were lost to follow-up, such that the utility of the education may have been overemphasized because the lost patients might have rejected the intervention. However, the marked improvements in inhaler handling, adherence, and satisfaction that we observed emphasize that education is useful. Second, other factors known to affect quality of life in COPD, such as the type of inhaler and the comorbidities, were not included in this study, Finally, we did not explore how long the effects of education persisted; more studies are needed on this topic.

This study also had several strengths. First, few such studies have been performed in Korea^27,37^; also, we enrolled only COPD patients; COPD and asthma differ, so the effects of education may also differ between these populations. Second, we assessed many quality of life outcomes (using the mMRC, CAT, EQ-5D, and PHQ-9 instruments), as well as inhaler satisfaction (using the FSI-10), and inhaler technique and adherence. As mentioned above, few studies have explored changes in quality of life after educational interventions. And to the best of our knowledge, this is the first study to report improved inhaler satisfaction after education. Improvements in inhaler satisfaction can lead to improvements in various clinical outcomes in COPD patients over the long time. This study highlights once again the importance of repeated inhaler education. Third, our study is different from other studies in that we have assessed the effects over a relatively long period of time (6 months). An assessment of how long the effects of education last can give the answer to how often education should be implemented. Our research is unique in this respect. Fourth, we found that the inhaler usage training was highly effective to improve inhaler satisfaction, technique, and adherence in a real-world setting, and that the effects were relatively persistent. In future studies, we will seek to precisely determine how long the effects of education persist.

## Conclusion

Repeated education delivered by an advanced practice nurse improved inhaler satisfaction, technique, and adherence. However, inhaler education did not significantly improve quality of life. More detailed studies are needed to determine the number of educational sessions required, the optimal intervals, and the duration of any benefits thus achieved.
